# Tricyclo­hex­yl{2-[(3,5-di-*tert*-butyl-4-hy­droxy­benz­yl)sulfan­yl]acetato-κ*O*}tin(IV)

**DOI:** 10.1107/S1600536810021884

**Published:** 2010-06-16

**Authors:** See Mun Lee, Hapipah Mohd Ali, Kong Mun Lo

**Affiliations:** a Department of Chemistry, University of Malaya, 50603 Kuala Lumpur, Malaysia

## Abstract

The title compound, [Sn(C_6_H_11_)_3_(C_17_H_25_O_3_S)], exists as a monomeric mol­ecule with the Sn^IV^ atom in a distorted tetra­hedral C_3_O coordination geometry. The presence of two bulky *tert*-butyl groups on the carboxyl­ate prevents any hydrogen-bonding inter­actions involving the hy­droxy group.

## Related literature

For tricyclo­hexyl­tin carboxyl­ates, see: Tiekink (1991 ▶). For a triphenyl­tin analogue of the title compound, see: Lee *et al.* (2009 ▶). For other related structures, see: Alcock & Timms (1968 ▶); Keng *et al.* (2010 ▶); Ng & Kumar Das (1997 ▶); Thong *et al.* (2009 ▶); Zhang *et al.* (2007 ▶). For the preparation of the ligand, see: Yehye *et al.* (2009 ▶).
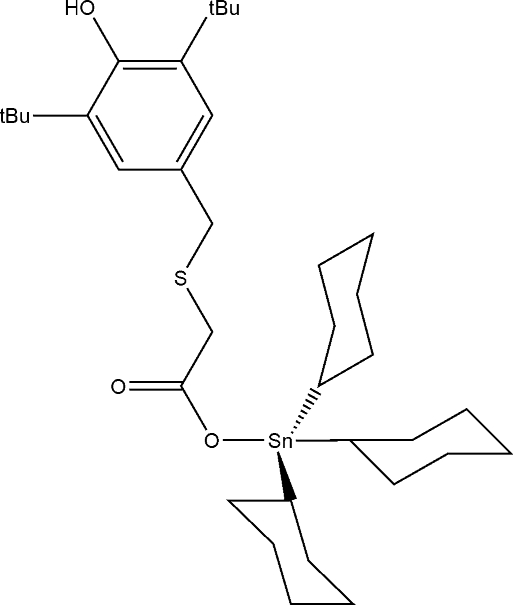


## Experimental

### 

#### Crystal data

[Sn(C_6_H_11_)_3_(C_17_H_25_O_3_S)]
*M* *_r_* = 677.56
Monoclinic, 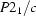

*a* = 15.5048 (3) Å
*b* = 11.4261 (3) Å
*c* = 19.9794 (4) Å
β = 94.603 (2)°
*V* = 3528.12 (14) Å^3^
*Z* = 4
Mo *K*α radiation
μ = 0.81 mm^−1^
*T* = 296 K
0.23 × 0.16 × 0.12 mm

#### Data collection

Bruker APEXII CCD diffractometer
Absorption correction: multi-scan (*SADABS*; Bruker, 2001 ▶) *T*_min_ = 0.835, *T*_max_ = 0.909
32619 measured reflections
8086 independent reflections
4695 reflections with *I* > 2σ(*I*)
*R*_int_ = 0.068

#### Refinement

*R*[*F* ^2^ > 2σ(*F* ^2^)] = 0.050
*wR*(*F* ^2^) = 0.117
*S* = 1.00
8086 reflections
368 parameters
H-atom parameters constrained
Δρ_max_ = 0.65 e Å^−3^
Δρ_min_ = −0.37 e Å^−3^

### 

Data collection: *APEX2* (Bruker, 2007 ▶); cell refinement: *SAINT* (Bruker, 2007 ▶); data reduction: *SAINT*; program(s) used to solve structure: *SHELXS97* (Sheldrick, 2008 ▶); program(s) used to refine structure: *SHELXL97* (Sheldrick, 2008 ▶); molecular graphics: *X-SEED* (Barbour, 2001 ▶); software used to prepare material for publication: *publCIF* (Westrip, 2010 ▶).

## Supplementary Material

Crystal structure: contains datablocks I, global. DOI: 10.1107/S1600536810021884/hy2317sup1.cif

Structure factors: contains datablocks I. DOI: 10.1107/S1600536810021884/hy2317Isup2.hkl

Additional supplementary materials:  crystallographic information; 3D view; checkCIF report

## Figures and Tables

**Table 1:** Selected bond lengths (Å)

Sn1—O2	2.081 (3)
Sn1—C18	2.148 (4)
Sn1—C24	2.157 (5)
Sn1—C30	2.166 (4)

**Fig. 1.**
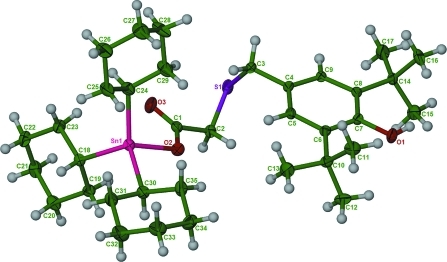
The molecular structure of the title compound, showing the 30% probability displacement ellipsoids.

## supplementary crystallographic information

### Comment

Triorganotin carboxylates are either monomeric or polymeric, depending primarily on the types of organic groups bonded to the tin atom. Triphenyltin carboxylates generally adopt a five-coordinated tin geometry with carboxylate bridges linking adjacent molecules into a polymeric chain, whereas tricyclohexyltin carboxylates have discrete four-coordinated tin structures (Tiekink, 1991).

The title compound is another example of a four-coordinated tricyclohexyltin carboxylate, in which the Sn^IV^ atom is in a distorted tetrahedral geometry. The close proximity of the carboxylate O3 towards the Sn atom [Sn1···O3 = 2.897 (3) Å] contributes to the distortion of the geometry (Alcock & Timms, 1968).

### Experimental

The title compound was prepared by refluxing 2-(3,5-di-*tert*-4-hydroxybenzyl)sulfanylacetic acid (0.39 g, 1 mmol) with tricyclohexyltin hydroxide (0.39 g, 1 mmol) in absolute ethanol for 2 h. Colourless crystals were obtained by slow evaporation of the solution at room temperature.

### Refinement

H atoms were placed at calculated positions and treated as riding on their parent atoms, with C—H = 0.93 (aromatic), 0.98 (CH), 0.97 (CH_2_) and 0.96 (CH_3_) Å and O—H = 0.82 Å and with *U*_iso_(H) = 1.2(1.5 for methyl and hydroxyl)*U*_eq_(C,O).

### Crystal data

**Table:**

[Sn(C_6_H_11_)_3_(C_17_H_25_O_3_S)]	*F*(000) = 1432
*M**_r_* = 677.56	*D*_x_ = 1.276 Mg m^−^^3^
Monoclinic, *P*2_1_/*c*	Mo *K*α radiation, λ = 0.71073 Å
Hall symbol: -P 2ybc	Cell parameters from 4134 reflections
*a* = 15.5048 (3) Å	θ = 2.2–20.5°
*b* = 11.4261 (3) Å	µ = 0.81 mm^−^^1^
*c* = 19.9794 (4) Å	*T* = 296 K
β = 94.603 (2)°	Prism, colourless
*V* = 3528.12 (14) Å^3^	0.23 × 0.16 × 0.12 mm
*Z* = 4	

### Data collection

**Table:**

Bruker APEXII CCD diffractometer	8086 independent reflections
Radiation source: fine-focus sealed tube	4695 reflections with *I* > 2σ(*I*)
graphite	*R*_int_ = 0.068
φ and ω scans	θ_max_ = 27.5°, θ_min_ = 1.3°
Absorption correction: multi-scan (*SADABS*; Bruker, 2001)	*h* = −20→20
*T*_min_ = 0.835, *T*_max_ = 0.909	*k* = −14→14
32619 measured reflections	*l* = −25→25

### Refinement

**Table:**

Refinement on *F*^2^	Primary atom site location: structure-invariant direct methods
Least-squares matrix: full	Secondary atom site location: difference Fourier map
*R*[*F*^2^ > 2σ(*F*^2^)] = 0.050	Hydrogen site location: inferred from neighbouring sites
*wR*(*F*^2^) = 0.117	H-atom parameters constrained
*S* = 1.00	*w* = 1/[σ^2^(*F*_o_^2^) + (0.0472*P*)^2^ + 1.0822*P*] where *P* = (*F*_o_^2^ + 2*F*_c_^2^)/3
8086 reflections	(Δ/σ)_max_ = 0.002
368 parameters	Δρ_max_ = 0.65 e Å^−^^3^
0 restraints	Δρ_min_ = −0.37 e Å^−^^3^

### Fractional atomic coordinates and isotropic or equivalent isotropic displacement parameters (Å^2^)

**Table:**

	*x*	*y*	*z*	*U*_iso_*/*U*_eq_	
Sn1	0.675022 (18)	0.68259 (2)	0.423505 (14)	0.05384 (11)	
S1	0.91268 (8)	0.32747 (11)	0.47742 (6)	0.0688 (3)	
O1	0.81458 (19)	0.1966 (3)	0.78627 (15)	0.0712 (9)	
H1	0.8495	0.1485	0.8025	0.107*	
O2	0.70824 (19)	0.5181 (3)	0.46211 (15)	0.0656 (8)	
C1	0.7760 (3)	0.4750 (4)	0.4374 (2)	0.0571 (10)	
O3	0.8166 (2)	0.5278 (3)	0.39778 (18)	0.0897 (11)	
C2	0.7988 (3)	0.3518 (3)	0.4606 (2)	0.0570 (11)	
H2A	0.7766	0.2970	0.4263	0.068*	
H2B	0.7703	0.3355	0.5010	0.068*	
C3	0.9325 (3)	0.4088 (4)	0.5546 (2)	0.0637 (12)	
H3A	0.9943	0.4217	0.5628	0.076*	
H3B	0.9050	0.4848	0.5491	0.076*	
C4	0.9002 (3)	0.3496 (3)	0.6156 (2)	0.0512 (10)	
C9	0.9478 (2)	0.2609 (4)	0.64754 (19)	0.0500 (9)	
H9	0.9989	0.2374	0.6302	0.060*	
C8	0.9223 (2)	0.2053 (3)	0.70464 (19)	0.0474 (9)	
C7	0.8447 (3)	0.2435 (4)	0.72873 (19)	0.0503 (9)	
C6	0.7929 (2)	0.3316 (3)	0.6972 (2)	0.0508 (10)	
C5	0.8240 (3)	0.3836 (3)	0.6404 (2)	0.0527 (10)	
H5	0.7920	0.4432	0.6187	0.063*	
C10	0.7064 (3)	0.3702 (4)	0.7228 (2)	0.0593 (11)	
C11	0.7205 (3)	0.4203 (4)	0.7944 (2)	0.0813 (15)	
H11A	0.7474	0.3621	0.8238	0.122*	
H11B	0.6658	0.4420	0.8099	0.122*	
H11C	0.7572	0.4879	0.7940	0.122*	
C13	0.6623 (3)	0.4652 (4)	0.6783 (3)	0.0890 (16)	
H13A	0.7001	0.5315	0.6768	0.134*	
H13B	0.6095	0.4886	0.6965	0.134*	
H13C	0.6496	0.4351	0.6337	0.134*	
C12	0.6446 (3)	0.2650 (5)	0.7238 (3)	0.0836 (15)	
H12A	0.6377	0.2304	0.6799	0.125*	
H12B	0.5893	0.2909	0.7366	0.125*	
H12C	0.6681	0.2081	0.7556	0.125*	
C14	0.9797 (3)	0.1098 (4)	0.7400 (2)	0.0549 (10)	
C16	1.0166 (4)	0.1528 (5)	0.8085 (2)	0.0901 (16)	
H16A	1.0515	0.0925	0.8303	0.135*	
H16B	0.9700	0.1721	0.8355	0.135*	
H16C	1.0514	0.2211	0.8030	0.135*	
C15	0.9297 (3)	−0.0044 (4)	0.7481 (3)	0.0838 (15)	
H15A	0.8964	−0.0224	0.7068	0.126*	
H15B	0.8916	0.0045	0.7834	0.126*	
H15C	0.9698	−0.0668	0.7592	0.126*	
C17	1.0558 (3)	0.0774 (5)	0.6999 (3)	0.0928 (18)	
H17A	1.0345	0.0500	0.6563	0.139*	
H17B	1.0891	0.0167	0.7230	0.139*	
H17C	1.0916	0.1449	0.6953	0.139*	
C18	0.6470 (3)	0.6709 (4)	0.31668 (19)	0.0547 (10)	
H18	0.6030	0.7304	0.3047	0.066*	
C23	0.7229 (3)	0.6982 (4)	0.2761 (2)	0.0698 (12)	
H23A	0.7700	0.6449	0.2891	0.084*	
H23B	0.7428	0.7771	0.2863	0.084*	
C22	0.6995 (4)	0.6874 (4)	0.2010 (2)	0.0835 (15)	
H22A	0.6588	0.7488	0.1867	0.100*	
H22B	0.7511	0.6980	0.1773	0.100*	
C21	0.6598 (4)	0.5699 (5)	0.1829 (2)	0.0835 (15)	
H21A	0.6426	0.5680	0.1351	0.100*	
H21B	0.7027	0.5091	0.1925	0.100*	
C20	0.5826 (3)	0.5459 (5)	0.2212 (2)	0.0797 (14)	
H20A	0.5604	0.4684	0.2100	0.096*	
H20B	0.5375	0.6023	0.2084	0.096*	
C19	0.6063 (3)	0.5537 (4)	0.2967 (2)	0.0658 (12)	
H19A	0.5547	0.5425	0.3203	0.079*	
H19B	0.6466	0.4914	0.3101	0.079*	
C30	0.5538 (3)	0.6957 (4)	0.4699 (2)	0.0628 (11)	
H30	0.5175	0.6308	0.4523	0.075*	
C35	0.5628 (3)	0.6826 (5)	0.5445 (2)	0.0833 (15)	
H35A	0.6024	0.7418	0.5635	0.100*	
H35B	0.5876	0.6066	0.5558	0.100*	
C31	0.5065 (3)	0.8080 (4)	0.4489 (2)	0.0790 (14)	
H31A	0.4963	0.8100	0.4004	0.095*	
H31B	0.5423	0.8747	0.4628	0.095*	
C24	0.7751 (3)	0.8051 (4)	0.4572 (3)	0.0741 (13)	
H24	0.8144	0.8078	0.4212	0.089*	
C25	0.7395 (4)	0.9250 (4)	0.4611 (3)	0.0963 (18)	
H25A	0.6952	0.9252	0.4928	0.116*	
H25B	0.7122	0.9464	0.4175	0.116*	
C32	0.4200 (3)	0.8167 (6)	0.4804 (3)	0.104 (2)	
H32A	0.3930	0.8910	0.4681	0.125*	
H32B	0.3820	0.7549	0.4625	0.125*	
C29	0.8294 (4)	0.7719 (5)	0.5180 (3)	0.1028 (19)	
H29A	0.8588	0.6989	0.5099	0.123*	
H29B	0.7928	0.7591	0.5545	0.123*	
C28	0.8968 (4)	0.8659 (5)	0.5389 (4)	0.119 (2)	
H28A	0.9251	0.8454	0.5823	0.142*	
H28B	0.9405	0.8667	0.5067	0.142*	
C27	0.8600 (4)	0.9832 (5)	0.5428 (3)	0.115 (2)	
H27A	0.8249	0.9866	0.5808	0.138*	
H27B	0.9066	1.0393	0.5506	0.138*	
C26	0.8072 (4)	1.0163 (5)	0.4826 (4)	0.116 (2)	
H26A	0.8444	1.0284	0.4465	0.139*	
H26B	0.7785	1.0898	0.4906	0.139*	
C33	0.4306 (3)	0.8070 (6)	0.5543 (3)	0.0961 (18)	
H33A	0.3742	0.8091	0.5721	0.115*	
H33B	0.4638	0.8731	0.5727	0.115*	
C34	0.4763 (4)	0.6944 (5)	0.5756 (3)	0.0945 (17)	
H34A	0.4396	0.6284	0.5620	0.113*	
H34B	0.4862	0.6927	0.6242	0.113*	

### Atomic displacement parameters (Å^2^)

**Table:**

	*U*^11^	*U*^22^	*U*^33^	*U*^12^	*U*^13^	*U*^23^
Sn1	0.05522 (17)	0.05444 (18)	0.05190 (18)	0.00493 (15)	0.00459 (12)	−0.00436 (15)
S1	0.0727 (7)	0.0773 (8)	0.0591 (7)	0.0236 (7)	0.0228 (6)	0.0165 (6)
O1	0.0712 (19)	0.082 (2)	0.0642 (19)	0.0205 (16)	0.0254 (16)	0.0273 (16)
O2	0.0657 (18)	0.0605 (19)	0.072 (2)	0.0070 (15)	0.0132 (15)	−0.0017 (15)
C1	0.055 (3)	0.060 (3)	0.056 (3)	0.000 (2)	−0.005 (2)	0.002 (2)
O3	0.086 (2)	0.085 (2)	0.102 (3)	0.0218 (19)	0.031 (2)	0.043 (2)
C2	0.068 (3)	0.048 (2)	0.054 (3)	0.001 (2)	0.004 (2)	−0.0021 (19)
C3	0.056 (3)	0.071 (3)	0.064 (3)	−0.012 (2)	0.004 (2)	0.018 (2)
C4	0.054 (2)	0.047 (2)	0.052 (2)	−0.0078 (19)	0.0037 (19)	0.0063 (18)
C9	0.046 (2)	0.055 (2)	0.049 (2)	0.0007 (19)	0.0048 (18)	0.006 (2)
C8	0.048 (2)	0.048 (2)	0.046 (2)	−0.0017 (18)	−0.0007 (17)	0.0014 (18)
C7	0.053 (2)	0.051 (2)	0.047 (2)	0.000 (2)	0.0099 (18)	0.0037 (19)
C6	0.052 (2)	0.044 (2)	0.056 (2)	−0.0022 (19)	0.0043 (18)	0.0001 (19)
C5	0.057 (2)	0.043 (2)	0.056 (3)	0.001 (2)	−0.004 (2)	0.0075 (19)
C10	0.054 (2)	0.052 (2)	0.073 (3)	0.002 (2)	0.010 (2)	0.000 (2)
C11	0.072 (3)	0.089 (4)	0.085 (4)	0.006 (3)	0.021 (3)	−0.020 (3)
C13	0.070 (3)	0.080 (4)	0.117 (4)	0.025 (3)	0.009 (3)	0.019 (3)
C12	0.053 (3)	0.077 (3)	0.123 (4)	−0.007 (3)	0.015 (3)	−0.004 (3)
C14	0.054 (2)	0.057 (3)	0.054 (3)	0.007 (2)	0.0040 (19)	0.011 (2)
C16	0.104 (4)	0.099 (4)	0.063 (3)	0.010 (3)	−0.022 (3)	0.013 (3)
C15	0.088 (4)	0.059 (3)	0.104 (4)	0.008 (3)	0.004 (3)	0.017 (3)
C17	0.065 (3)	0.116 (5)	0.099 (4)	0.038 (3)	0.020 (3)	0.033 (3)
C18	0.058 (2)	0.056 (3)	0.050 (2)	0.009 (2)	0.0044 (18)	−0.003 (2)
C23	0.081 (3)	0.067 (3)	0.063 (3)	−0.007 (2)	0.009 (2)	0.001 (2)
C22	0.112 (4)	0.081 (4)	0.059 (3)	−0.007 (3)	0.016 (3)	0.011 (3)
C21	0.116 (4)	0.082 (4)	0.053 (3)	0.014 (3)	0.010 (3)	−0.007 (3)
C20	0.089 (4)	0.080 (3)	0.067 (3)	0.006 (3)	−0.012 (3)	−0.018 (3)
C19	0.064 (3)	0.072 (3)	0.062 (3)	−0.005 (2)	0.005 (2)	−0.002 (2)
C30	0.061 (3)	0.070 (3)	0.059 (3)	0.007 (2)	0.011 (2)	−0.005 (2)
C35	0.085 (3)	0.099 (4)	0.066 (3)	0.020 (3)	0.013 (3)	0.016 (3)
C31	0.068 (3)	0.096 (4)	0.073 (3)	0.020 (3)	0.010 (2)	0.010 (3)
C24	0.073 (3)	0.068 (3)	0.078 (3)	−0.001 (3)	−0.009 (2)	−0.008 (3)
C25	0.096 (4)	0.056 (3)	0.129 (5)	−0.008 (3)	−0.039 (3)	0.017 (3)
C32	0.073 (3)	0.136 (6)	0.105 (5)	0.034 (4)	0.017 (3)	−0.007 (4)
C29	0.110 (4)	0.062 (3)	0.127 (5)	0.010 (3)	−0.044 (4)	−0.002 (3)
C28	0.094 (4)	0.085 (4)	0.164 (6)	0.008 (4)	−0.066 (4)	−0.015 (4)
C27	0.133 (5)	0.082 (4)	0.121 (5)	−0.014 (4)	−0.036 (4)	−0.018 (4)
C26	0.113 (5)	0.060 (3)	0.164 (6)	−0.015 (3)	−0.045 (4)	0.011 (4)
C33	0.076 (3)	0.121 (5)	0.095 (4)	0.009 (3)	0.026 (3)	−0.023 (4)
C34	0.097 (4)	0.113 (5)	0.077 (4)	−0.015 (4)	0.037 (3)	0.002 (3)

### Geometric parameters (Å, °)

**Table:**

Sn1—O2	2.081 (3)	C18—C19	1.520 (6)
Sn1—C18	2.148 (4)	C18—H18	0.9800
Sn1—C24	2.157 (5)	C23—C22	1.521 (6)
Sn1—C30	2.166 (4)	C23—H23A	0.9700
S1—C2	1.792 (4)	C23—H23B	0.9700
S1—C3	1.806 (5)	C22—C21	1.509 (6)
O1—C7	1.383 (4)	C22—H22A	0.9700
O1—H1	0.8200	C22—H22B	0.9700
O2—C1	1.294 (5)	C21—C20	1.497 (7)
C1—O3	1.211 (5)	C21—H21A	0.9700
C1—C2	1.515 (6)	C21—H21B	0.9700
C2—H2A	0.9700	C20—C19	1.526 (6)
C2—H2B	0.9700	C20—H20A	0.9700
C3—C4	1.514 (5)	C20—H20B	0.9700
C3—H3A	0.9700	C19—H19A	0.9700
C3—H3B	0.9700	C19—H19B	0.9700
C4—C5	1.373 (5)	C30—C35	1.493 (6)
C4—C9	1.380 (5)	C30—C31	1.520 (6)
C9—C8	1.390 (5)	C30—H30	0.9800
C9—H9	0.9300	C35—C34	1.530 (7)
C8—C7	1.401 (5)	C35—H35A	0.9700
C8—C14	1.542 (5)	C35—H35B	0.9700
C7—C6	1.405 (5)	C31—C32	1.529 (6)
C6—C5	1.400 (5)	C31—H31A	0.9700
C6—C10	1.538 (5)	C31—H31B	0.9700
C5—H5	0.9300	C24—C29	1.471 (7)
C10—C13	1.529 (6)	C24—C25	1.481 (6)
C10—C12	1.538 (6)	C24—H24	0.9800
C10—C11	1.540 (6)	C25—C26	1.517 (7)
C11—H11A	0.9600	C25—H25A	0.9700
C11—H11B	0.9600	C25—H25B	0.9700
C11—H11C	0.9600	C32—C33	1.478 (7)
C13—H13A	0.9600	C32—H32A	0.9700
C13—H13B	0.9600	C32—H32B	0.9700
C13—H13C	0.9600	C29—C28	1.533 (8)
C12—H12A	0.9600	C29—H29A	0.9700
C12—H12B	0.9600	C29—H29B	0.9700
C12—H12C	0.9600	C28—C27	1.461 (8)
C14—C16	1.521 (6)	C28—H28A	0.9700
C14—C17	1.524 (6)	C28—H28B	0.9700
C14—C15	1.533 (6)	C27—C26	1.449 (7)
C16—H16A	0.9600	C27—H27A	0.9700
C16—H16B	0.9600	C27—H27B	0.9700
C16—H16C	0.9600	C26—H26A	0.9700
C15—H15A	0.9600	C26—H26B	0.9700
C15—H15B	0.9600	C33—C34	1.513 (7)
C15—H15C	0.9600	C33—H33A	0.9700
C17—H17A	0.9600	C33—H33B	0.9700
C17—H17B	0.9600	C34—H34A	0.9700
C17—H17C	0.9600	C34—H34B	0.9700
C18—C23	1.513 (6)		
			
O2—Sn1—C18	109.65 (13)	C18—C23—H23B	109.2
O2—Sn1—C24	108.65 (15)	C22—C23—H23B	109.2
C18—Sn1—C24	115.72 (18)	H23A—C23—H23B	107.9
O2—Sn1—C30	95.82 (14)	C21—C22—C23	111.7 (4)
C18—Sn1—C30	108.43 (16)	C21—C22—H22A	109.3
C24—Sn1—C30	116.65 (18)	C23—C22—H22A	109.3
C2—S1—C3	100.3 (2)	C21—C22—H22B	109.3
C7—O1—H1	109.5	C23—C22—H22B	109.3
C1—O2—Sn1	112.7 (3)	H22A—C22—H22B	107.9
O3—C1—O2	122.7 (4)	C20—C21—C22	111.5 (4)
O3—C1—C2	122.8 (4)	C20—C21—H21A	109.3
O2—C1—C2	114.4 (4)	C22—C21—H21A	109.3
C1—C2—S1	113.8 (3)	C20—C21—H21B	109.3
C1—C2—H2A	108.8	C22—C21—H21B	109.3
S1—C2—H2A	108.8	H21A—C21—H21B	108.0
C1—C2—H2B	108.8	C21—C20—C19	110.8 (4)
S1—C2—H2B	108.8	C21—C20—H20A	109.5
H2A—C2—H2B	107.7	C19—C20—H20A	109.5
C4—C3—S1	114.4 (3)	C21—C20—H20B	109.5
C4—C3—H3A	108.7	C19—C20—H20B	109.5
S1—C3—H3A	108.7	H20A—C20—H20B	108.1
C4—C3—H3B	108.7	C18—C19—C20	111.9 (4)
S1—C3—H3B	108.7	C18—C19—H19A	109.2
H3A—C3—H3B	107.6	C20—C19—H19A	109.2
C5—C4—C9	119.0 (4)	C18—C19—H19B	109.2
C5—C4—C3	121.0 (4)	C20—C19—H19B	109.2
C9—C4—C3	120.1 (4)	H19A—C19—H19B	107.9
C4—C9—C8	122.6 (4)	C35—C30—C31	111.3 (4)
C4—C9—H9	118.7	C35—C30—Sn1	113.9 (3)
C8—C9—H9	118.7	C31—C30—Sn1	110.9 (3)
C9—C8—C7	116.6 (3)	C35—C30—H30	106.7
C9—C8—C14	120.5 (3)	C31—C30—H30	106.7
C7—C8—C14	122.9 (3)	Sn1—C30—H30	106.7
O1—C7—C8	121.4 (3)	C30—C35—C34	112.5 (4)
O1—C7—C6	115.6 (3)	C30—C35—H35A	109.1
C8—C7—C6	123.0 (3)	C34—C35—H35A	109.1
C5—C6—C7	116.4 (3)	C30—C35—H35B	109.1
C5—C6—C10	120.9 (4)	C34—C35—H35B	109.1
C7—C6—C10	122.6 (3)	H35A—C35—H35B	107.8
C4—C5—C6	122.4 (4)	C30—C31—C32	111.2 (4)
C4—C5—H5	118.8	C30—C31—H31A	109.4
C6—C5—H5	118.8	C32—C31—H31A	109.4
C13—C10—C6	111.8 (4)	C30—C31—H31B	109.4
C13—C10—C12	108.2 (4)	C32—C31—H31B	109.4
C6—C10—C12	110.0 (4)	H31A—C31—H31B	108.0
C13—C10—C11	107.4 (4)	C29—C24—C25	112.7 (4)
C6—C10—C11	110.6 (3)	C29—C24—Sn1	116.1 (4)
C12—C10—C11	108.7 (4)	C25—C24—Sn1	110.8 (3)
C10—C11—H11A	109.5	C29—C24—H24	105.4
C10—C11—H11B	109.5	C25—C24—H24	105.4
H11A—C11—H11B	109.5	Sn1—C24—H24	105.4
C10—C11—H11C	109.5	C24—C25—C26	113.5 (5)
H11A—C11—H11C	109.5	C24—C25—H25A	108.9
H11B—C11—H11C	109.5	C26—C25—H25A	108.9
C10—C13—H13A	109.5	C24—C25—H25B	108.9
C10—C13—H13B	109.5	C26—C25—H25B	108.9
H13A—C13—H13B	109.5	H25A—C25—H25B	107.7
C10—C13—H13C	109.5	C33—C32—C31	112.1 (5)
H13A—C13—H13C	109.5	C33—C32—H32A	109.2
H13B—C13—H13C	109.5	C31—C32—H32A	109.2
C10—C12—H12A	109.5	C33—C32—H32B	109.2
C10—C12—H12B	109.5	C31—C32—H32B	109.2
H12A—C12—H12B	109.5	H32A—C32—H32B	107.9
C10—C12—H12C	109.5	C24—C29—C28	112.1 (5)
H12A—C12—H12C	109.5	C24—C29—H29A	109.2
H12B—C12—H12C	109.5	C28—C29—H29A	109.2
C16—C14—C17	107.5 (4)	C24—C29—H29B	109.2
C16—C14—C15	109.7 (4)	C28—C29—H29B	109.2
C17—C14—C15	105.5 (4)	H29A—C29—H29B	107.9
C16—C14—C8	110.2 (4)	C27—C28—C29	113.4 (5)
C17—C14—C8	111.9 (3)	C27—C28—H28A	108.9
C15—C14—C8	111.9 (3)	C29—C28—H28A	108.9
C14—C16—H16A	109.5	C27—C28—H28B	108.9
C14—C16—H16B	109.5	C29—C28—H28B	108.9
H16A—C16—H16B	109.5	H28A—C28—H28B	107.7
C14—C16—H16C	109.5	C26—C27—C28	113.0 (5)
H16A—C16—H16C	109.5	C26—C27—H27A	109.0
H16B—C16—H16C	109.5	C28—C27—H27A	109.0
C14—C15—H15A	109.5	C26—C27—H27B	109.0
C14—C15—H15B	109.5	C28—C27—H27B	109.0
H15A—C15—H15B	109.5	H27A—C27—H27B	107.8
C14—C15—H15C	109.5	C27—C26—C25	112.8 (5)
H15A—C15—H15C	109.5	C27—C26—H26A	109.0
H15B—C15—H15C	109.5	C25—C26—H26A	109.0
C14—C17—H17A	109.5	C27—C26—H26B	109.0
C14—C17—H17B	109.5	C25—C26—H26B	109.0
H17A—C17—H17B	109.5	H26A—C26—H26B	107.8
C14—C17—H17C	109.5	C32—C33—C34	110.9 (5)
H17A—C17—H17C	109.5	C32—C33—H33A	109.5
H17B—C17—H17C	109.5	C34—C33—H33A	109.5
C23—C18—C19	111.4 (3)	C32—C33—H33B	109.5
C23—C18—Sn1	114.5 (3)	C34—C33—H33B	109.5
C19—C18—Sn1	111.3 (3)	H33A—C33—H33B	108.1
C23—C18—H18	106.4	C33—C34—C35	111.5 (4)
C19—C18—H18	106.4	C33—C34—H34A	109.3
Sn1—C18—H18	106.4	C35—C34—H34A	109.3
C18—C23—C22	112.2 (4)	C33—C34—H34B	109.3
C18—C23—H23A	109.2	C35—C34—H34B	109.3
C22—C23—H23A	109.2	H34A—C34—H34B	108.0
			
C18—Sn1—O2—C1	60.5 (3)	C30—Sn1—C18—C23	162.2 (3)
C24—Sn1—O2—C1	−66.9 (3)	O2—Sn1—C18—C19	33.1 (3)
C30—Sn1—O2—C1	172.5 (3)	C24—Sn1—C18—C19	156.4 (3)
Sn1—O2—C1—O3	2.4 (5)	C30—Sn1—C18—C19	−70.4 (3)
Sn1—O2—C1—C2	−176.0 (3)	C19—C18—C23—C22	52.1 (5)
O3—C1—C2—S1	41.6 (6)	Sn1—C18—C23—C22	179.5 (3)
O2—C1—C2—S1	−140.0 (3)	C18—C23—C22—C21	−53.4 (6)
C3—S1—C2—C1	72.6 (3)	C23—C22—C21—C20	55.6 (6)
C2—S1—C3—C4	74.6 (3)	C22—C21—C20—C19	−56.4 (6)
S1—C3—C4—C5	−101.0 (4)	C23—C18—C19—C20	−53.3 (5)
S1—C3—C4—C9	79.7 (4)	Sn1—C18—C19—C20	177.6 (3)
C5—C4—C9—C8	−0.9 (6)	C21—C20—C19—C18	55.5 (5)
C3—C4—C9—C8	178.4 (4)	O2—Sn1—C30—C35	56.5 (4)
C4—C9—C8—C7	0.2 (6)	C18—Sn1—C30—C35	169.5 (3)
C4—C9—C8—C14	−177.6 (4)	C24—Sn1—C30—C35	−57.8 (4)
C9—C8—C7—O1	−178.1 (4)	O2—Sn1—C30—C31	−176.9 (3)
C14—C8—C7—O1	−0.3 (6)	C18—Sn1—C30—C31	−64.0 (3)
C9—C8—C7—C6	1.3 (6)	C24—Sn1—C30—C31	68.8 (4)
C14—C8—C7—C6	179.0 (4)	C31—C30—C35—C34	52.7 (6)
O1—C7—C6—C5	177.5 (4)	Sn1—C30—C35—C34	179.1 (4)
C8—C7—C6—C5	−1.9 (6)	C35—C30—C31—C32	−53.2 (6)
O1—C7—C6—C10	−2.6 (6)	Sn1—C30—C31—C32	178.9 (4)
C8—C7—C6—C10	178.0 (4)	O2—Sn1—C24—C29	−22.1 (5)
C9—C4—C5—C6	0.2 (6)	C18—Sn1—C24—C29	−145.9 (4)
C3—C4—C5—C6	−179.2 (4)	C30—Sn1—C24—C29	84.7 (4)
C7—C6—C5—C4	1.2 (6)	O2—Sn1—C24—C25	−152.3 (4)
C10—C6—C5—C4	−178.7 (4)	C18—Sn1—C24—C25	83.9 (4)
C5—C6—C10—C13	1.4 (6)	C30—Sn1—C24—C25	−45.5 (4)
C7—C6—C10—C13	−178.6 (4)	C29—C24—C25—C26	49.7 (7)
C5—C6—C10—C12	121.6 (4)	Sn1—C24—C25—C26	−178.3 (4)
C7—C6—C10—C12	−58.3 (5)	C30—C31—C32—C33	55.7 (7)
C5—C6—C10—C11	−118.3 (4)	C25—C24—C29—C28	−49.2 (7)
C7—C6—C10—C11	61.7 (5)	Sn1—C24—C29—C28	−178.5 (4)
C9—C8—C14—C16	112.1 (4)	C24—C29—C28—C27	50.5 (8)
C7—C8—C14—C16	−65.6 (5)	C29—C28—C27—C26	−51.6 (9)
C9—C8—C14—C17	−7.5 (6)	C28—C27—C26—C25	51.1 (9)
C7—C8—C14—C17	174.8 (4)	C24—C25—C26—C27	−50.4 (8)
C9—C8—C14—C15	−125.6 (4)	C31—C32—C33—C34	−56.3 (7)
C7—C8—C14—C15	56.7 (5)	C32—C33—C34—C35	54.8 (7)
O2—Sn1—C18—C23	−94.4 (3)	C30—C35—C34—C33	−53.6 (6)
C24—Sn1—C18—C23	28.9 (4)		
